# First-Episode Psychosis care in Central Europe: What we have learned and what we still need to do

**DOI:** 10.1192/j.eurpsy.2022.85

**Published:** 2022-09-01

**Authors:** J. Réthelyi

**Affiliations:** Semmelweis University, Department Of Psychiatry And Psychotherapy, Budapest, Hungary

**Keywords:** early intervention, First Episode Psychosis, Central and Eastern Europe

## Abstract

First Episode Psychosis (FEP) treatment is a critical element of mental health-care systems, which has been shown to improve outcomes in this patient group. Due to divergent historical and political trajectories, countries of Central and Eastern Europe have taken a different course in the development of mental health-care. Among these differences is the less advanced level of present FEP treatment networks. Traditionally mental health-care is more inpatient based in these countries, with a lesser emphasis on specialized outpatient services, and early intervention programmes have been only launched during the last 15 years. Despite the lag of the development of FEP services, a variety of models has been already started in our countries. In my lecture I will review the literature about the Central and Esteran European region. I will also present good practices of how the existing early intervention programmes can be integrated into the traditional mental health-care systems.

**Disclosure:**

No significant relationships.

